# How paradata can illuminate technical, social and professional role changes between the Poverty in the UK (1967/1968) and Poverty and Social Exclusion in the UK (2012) surveys

**DOI:** 10.1007/s11135-016-0403-5

**Published:** 2016-08-29

**Authors:** Rosalind Edwards, Ann Phoenix, David Gordon, Karen Bell, Heather Elliott, Eldin Fahmy

**Affiliations:** 1 ESRC National Centre for Research Methods, University of Southampton, Southampton, UK; 2 Thomas Coram Research Unit, UCL Institute of Education, London, UK; 3 The Townsend Centre for International Poverty Research, University of Bristol, Bristol, UK

**Keywords:** Field interviewers, History of social surveys, Micro paradata, Peter Townsend, Poverty research

## Abstract

This article brings together analyses of the micro paradata ‘by-products’ from the 1967/1968 Poverty in the United Kingdom (PinUK) and 2012 Poverty and Social Exclusion in the UK (PSE) surveys to explore changes in the conditions of production over this 45 year period. We highlight technical, social and professional role continuities and changes, shaped by the institutionalisation of survey researchers, the professionalization of the field interviewer, and economisation. While there are similarities between the surveys in that field interviewers were and are at the bottom of the research hierarchy, we demonstrate an increasing segregation between the core research team and field interviewers. In PinUK the field interviewers are visible in the paper survey booklets; through their handwritten notes on codes and in written marginalia they can ‘talk’ to the central research team. In PSE they are absent from the computer mediated data, and from communication with the central team. We argue that, while there have been other benefits to field interviewers, their relational labour has become less visible in a shift from the exercise of observational judgement to an emphasis on standardisation. Yet, analyses of what field interviewers actually do show that they still need to deploy the same interpersonal skills and resourcefulness to secure and maintain interviews as they did 45 years previously.

## Introduction

Peter Townsend’s Poverty in the United Kingdom (PinUK) study, conducted 1967–1968, marked a key historical moment, as part of the ‘rediscovery’ of poverty (Townsend 1962). Initially working with Brian Abel-Smith, Townsend challenged top-down, absolute theorisations of poverty solely as low income levels. Instead he conceptualised poverty as a sociological phenomenon, requiring the criteria for measurement to be relative, grounded in and derived empirically from the habitual spending practices, possessions and social activities of the society concerned. Building on four exploratory qualitative studies of groups at risk of poverty carried out by his researchers: large families (Land), mothers alone (Marsden), unemployment (Sinfield) and sickness and disability (Veit-Wilson),^1^ Townsend established a series of indicators covering major areas of personal, household and social life. He then conducted a large national household survey, which was groundbreaking in demonstrating that poverty excludes people from being full members of society and focusing academic and social policy attention on the importance of scientific definitions of poverty. His innovative conception and measurement of poverty has remained relevant. Latterly, it has been refined by the ESRC-funded Poverty and Social Exclusion (PSE) in the UK 2012 survey (Gordon et al. 2013: Lansley and Mack 2015), which builds on previous academic surveys including PinUK (Townsend 1979)^2^ in order to advance the ‘state of the art’ of the theory and practice of poverty and social exclusion measurement.

As well as being groundbreaking in its approach to poverty, PinUK and its successors are part of moments in the history of social surveys and broader societal shifts. In terms of society, these shifts include a transition from an ‘élite’ towards a ‘mass and democratised’ society (Osborne and Rose 1999), where deference can no longer be expected by the middle and upper classes purely by virtue of their social status, there is an expectation (rhetorically at least) of equality and of ‘ordinary’ people’s opinions being sought and taken into account in the direction of society, treating them as ‘thoughtful and rational citizens of a democracy’ (*op cit.* p. 382). For social surveys, reflecting changes in social relations, the moment is one of a shift from such research as an quasi-amateur ‘gentlemanly’ pursuit of wealthy individuals focused on differentiating and classifying populations to an institutionalised, professionalised and technologised occupation concerned with the everyday population (Kent 1981; Morton-Williams 1993; Savage 2010). As Savage (2010) demonstrates, the history of method and research processes, and historical and cultural change throw significant light on each other. In this article, we explore changes in the research process since the PinUK survey by examining the micro paradata recorded on the PinUK questionnaires and considering the experience of data collection on the PSE. We draw on an innovative project that brought together analyses of paradata from the 1967/1968 PinUK and the 2012 PSE surveys to explore and reflect on shifts in the more general process of conducting social survey research over the 45 years between the two surveys, and their implications for the relations involved in survey data production. Our historical comparison highlights technical, social and professional role continuities and changes in the context of the three overlapping main processes that Ayrton (2016) has identified as characterizing social survey work since the post-war period: the ‘institutionalisation’ of survey researchers; the ‘professionalisation’ of field interviewers and ‘economisation’.

Converse (2009) suggests that, as part of a response to an increasingly complex society, survey researchers have been institutionalised. This has happened through consolidation as a trade (and incorporation into academic and survey-focused organisations, Bulmer et al. 1991; Osborne and Rose 1999). At the same time, the field interviewer has been professionalised as a technician, conducting interviews using computers, and there has been a metaphorical professionalisation of survey respondents through general familiarity with the interview as a motif of contemporary society and sense of self (Atkinson and Silverman 1997). These two developments have coincided with economic imperatives for methodological innovation through technology to help to constrain relentlessly rising survey costs (Wright and Marsden 2010). They show how technical and social changes are inextricably linked with changes in field interviewers’ professional role.

This paper explores the interplay of gains and losses that have resulted from these related shifts in social survey research on poverty in the United Kingdom. They have contributed to a shift away from researchers being foregrounded in social research, to the data and analyses being foregrounded (Savage 2010). This means that survey managers have more direct knowledge of how interviewers conduct interviews. Yet, to some extent, this gives less autonomy to field interviewers and allows fewer insights into the ways in which interviewers’ feelings and preoccupations affect their conduct of interviews. The fact that potential participants are now more familiar with surveys than in the 1960s and that social researchers now have to adhere to ethical codes also shifts the power relationships in the interview encounter. We consider the ways in which social shifts in the ethics of research make it less acceptable to ask some questions and more acceptable to ask others. We first briefly discuss the study that informs the paper, then technological changes in the conduct of social surveys before finally considering the iterative relationship between social change and social survey research over the last half century before drawing these issues together by discussing professional role changes.

## The historical comparative study

The historical comparison at the heart of our ESRC cross-investment project involved secondary analysis of the micro-paradata in Poverty in the UK 1967/1968 survey booklets compared with primary data analysis for the Poverty and Social Exclusion in the UK 2012 survey.^3^ This encompassed:

- Comparison of two forms of paradata: thematic analysis of anonymised transcripts of 23 PSE survey interviews audio-recorded for the purpose of analysing both what interviewers and interviewees say (from 8546 completed individual survey interviews^4^), and narrative analysis of one of these; and thematic analyses of the marginal micro-paradata in 69 booklets from the 3566 households surveyed in PinUK^5^ selected from particular geographical areas represented in the original study. Six of these booklets were then theoretically sampled for narrative analysis.
- Video interviews with 17 PinUK research team members and colleagues, including field interviewers conducted for the current study, which focused on the conduct of the 1960s survey, and academic and political reception of its findings. These PinUK interviewees were accessed through personal networks and snowballing, as well as Google and 192.com searches. Deductive qualitative analysis, moving back and forth between theory and data (Gilgun 2004), was used to inform analysis of the key themes.
- Two ‘Community of Interest’ discussion groups with researchers working on major contemporary surveys such as the Census, Millennium Cohort Survey and Understanding Society, and GfK NOP and PSE researchers.

Ontologically, the team viewed participants and researchers as meaning-making within particular socio-historical contexts and so as co-constructing the responses and by-products of the survey interviews. Both the surveys that inform the study reported here were rigorously conducted by well-trained and competent field interviewers. Most of the material produced by the interviewers for both studies that we have drawn on in our comparative analysis here was ‘immediate’: the marginal micro-data in the 1967/1968 PinUK survey booklets was written at the time of, or very soon after, the actual interviews, and the interview interactions for the 2012 PSE survey were audio-captured at the time. Other data are less immediate: the interviews with various members of the PinUK research team are recollections nearly half a century later, some vivid and some rather hazy, but nonetheless informative about the background to the survey data collection process at the time.

In what follows, we do not imply a developmental periodisation of the social survey or an idealisation of past surveys, nor do we provide indicators of change in the nature of survey interviews. Instead, we point to the benefits and constraints of these social, technical and professional changes for contemporary survey interviews. We aim to examine some examples from both surveys that throw light on the conditions of production in a period in the history of social surveys that has received limited attention (post-war) and compare these with contemporary conditions, in order to provide insights into how social surveys are reflective of and shaped by wider societal shifts.

## Technological changes in social survey research

Both PinUK and PSE involve mixed modes of data collection to the extent that, while each was administered face-to-face, some questions required visual choice by way of show cards. A major difference between the two surveys, however, is that the introduction of computer-assisted personal interviewing (CAPI) means that, instead of collecting data through paper questionnaires as for PinUK, data are now collected via laptop computers programmed with the survey. Field interviewers enter responses directly into the survey computer programme and ‘logic checks’ identify contradictory or inconsistent responses. Rather than having to take decisions about the order in which to move through the questionnaires, they are automatically routed through to the next appropriate survey question (which can be customised depending on the informant’s previous answers).

Couper (1998) advocated the use of by-product CAPI data (‘paradata’) to analyse the process by which survey data were collected, in order to evaluate, monitor and manage various survey processes. Since then, analyses of automatically generated information (e.g. time stamps, keystrokes, use of the help window) have burgeoned. Further, there is now electronic tracking and tracing of participants as well as computer power to process the data—a change that allowed us to search online for, and access by email, some of the original PinUK field interviewers and research team—unlike the painstaking processes that were entailed in the 1960s.

These technological changes coincided with fewer ‘housewives’ being available at home in the daytime to be surveyed, decreasing commitment from the general population to taking part as survey research lost its novelty value and deference in society declined, and questioning of the ways in which social researchers analysed and presented research participants (Crothers and Platt 2010; Rossi et al. 1983; Savage 2010). Analyses of paradata usually concern ways to evaluate and improve responses while reducing the high costs of survey data collection (Nicholaas 2011). The emphasis has, therefore, been on the monitoring of how field interviewers present questionnaires and an emphasis on consistency in the data collection process to improve the quality of surveys. In consequence, field interviewers are likely to be well aware that they are subject to surveillance and can be monitored online, in real time. Zuboff (1989) likens this to Foucault’s (1977) panopticon since the interviewers do not know when they are being monitored.

This ‘electronic leash’ (Barnett 1998) has implications for the field interviewer role that become clearer if the qualitative, marginal paradata from the 1967/1968 PinUK survey is compared with the paradata from the PSE interview transcripts. Training for survey field interviewers advanced significantly in the UK in the 1970s, signalled by the appearance of Atkinson’s (1971) classic manual: *Handbook for Interviewers* (Marsh 1982). In the PinUK survey, Townsend made detailed training materials on poverty available to the field interviewers and the instructions in the survey booklets are painstakingly comprehensive. But as several of the field interviewers we spoke to recounted, they knew that they were largely on their own to collect the required data once they were provided with their quota of booklets and list of addresses to visit. As one (IM) told us:

I had a car and I was given a list of addresses to go to. Not where I lived in Manchester but Shropshire was one … I know I went to a number of destinations … My impression was that you were left very much on your own, very much on your own.

This autonomy contrasts with the standardised and formalised briefing and training for the 2012 PSE survey, which (in keeping with current social survey practices) focused on using the interview toolkit (CAPI, showcards, shuffle cards, etc.), gaining access, following the interview script, uploading the survey answers each day, discussing how incentive payments should be explained, guidance about who are acceptable translators/interpreters where the interviewee does not speak English well and managing the self-completion section where the laptop needs to be handed back and forth, etc. Arguably these aspects of the survey process are deskilling in comparison with the independence given to the PinUK field researchers and are potentially stressful for contemporary field interviewers. The following example shows the anxiety that interviewers sometimes experience in attempting to establishing rapport while logging on to the computer survey and ensuring that it is working; a process that the technology could make more stressful than the pen and paper method of PinUK:

Interviewee: How many people will you be seeing tonight?

Interviewer: Oh, well if that computer’s giving me a bit of jip, I’ll, I’ll call it a draw. I still say there’s something wrong with it and they keep saying, oh it’s just the security. Every computer I’ve, the one before that was brilliant. It used to start up in no time and

Interviewee: Yeah.

Interviewer: Never had any trouble with it. This one keeps freezing.

Interviewee: Not fun is it?

In the following extract, the interviewer appears to use talk to settle herself into the interviewer role while trying to get through the password protection, which is more stringent than usual because of the PSE paradata project:

Interviewer: there we go. I’ve got a password, they sent them out, because we don’t usually do this for this particular research project.

Interviewee: Okay.

Interviewer: I had a little old lady, couldn’t get a password from anybody, all out the office [laughs] I’m sorry [laughs] we’ll carry on, I won’t record it. Okay, erm, okay I’ll give you these. … Okay, not through security yet.

We know from the earlier PinUK qualitative paradata written on the survey booklets, that some field interviewers were anxious about getting the necessary information. What was different from the PSE survey, however, was that the 1960s researchers were able to use marginal notes to justify not obtaining some responses or to amplify and explain what they had asked, the responses they had got or the codes they had selected. For example:

Brother owns shop but 2^nd^ [the husband was the second respondent in household, his wife was the first] seems to take what he makes out of it, therefore coded as employed. (Booklet 6/36 0239)

Some created micro paradata to debrief themselves or the team on the interviews and the circumstances in which they had carried them out. For example, one field interviewer informed the central research team in relation to a question to be asked of ‘housewives’ about whether and how often they felt tired:

I did not ask this question. 87 year old woman technically housewife, son does many duties also and it seemed foolish to ask 87 yr old if she felt tired! (Booklet 6/35 1759)

In current surveys, such as PSE, codes are automatically generated from the pre-coded responses selected, which may well remove some of the anxiety the PinUK interviewers sometimes felt about coding. However, the PSE transcripts show that interviewers did sometimes have difficulty in doing coding:

Interviewer: Damp, damp free home, where do you put that? Erm, it doesn’t actually go anywhere does it?

Interviewee: No.

Interviewer: ‘Cos you haven’t got it at the moment, but it’s not because you can’t afford it because it’s being done, I’ll leave it outside of those I think. Right, I’ll code these up.

Doubts about coding may be discernible in the PSE and other contemporary surveys through the keystroke time record (though there may be other reasons for delays in inputting data, such as interviewers taking time to think about codes or to read flash cards). It is possible for field interviewers to call up a free text box and insert comments about answers to specific questions, however this may be difficult to do in the middle of an interview as typing a long comment into a laptop after a respondent has given a ‘yes/no’ answer may appear suspicious and/or rude. Given that interview contexts are complex interactional spaces (Kvale 1996), it is also sometimes difficult to be certain what is happening when interviewers express doubt about coding to interviewees, as in the following extract from a PSE transcript:

Interviewer: … Last time I interviewed you you said you received help from friends and family as well, what impact would you say receiving this help has had on your material standard of living? Impact on current standard of living as opposed to the impact at the time of the original interview.

Interviewee: You mean has it got worse or has it got better? I don’t really understand the question.

Interviewer: No I don’t either [laughs]. We are trying to find out about any impact on the goods, oh the goods that you’re able to own… The fact they’re giving you money, does that have a big impact, a little impact or no impact at all?

Interviewee: No impact at all really, ‘cos you end up paying it back anyway, then you’re short that week…

Interviewer: I think that’s why, that’s why they’re saying that there’s no material impact, it’s just that…

Interviewee: No.

At first sight, it seems that both the field interviewer and interviewee were uncertain about the meaning of the question. Knowledge of the background to the interview, however, decentres this interpretation. The 23 PSE interviewers recorded in the current study were highly experienced, and the PSE followed-up respondents to the Family Resources Survey, with re-interviews usually conducted by the same interviewers. The above interviewer had previously asked several questions about giving and receiving gifts from/to friends and family. The codes provided the interviewer with the details of the kind of help the respondents could give/received from friends and family, while the computer screen also showed details about the meaning of the question, which also had been discussed in the field interviewer briefings. It seems likely then that the interviewer’s;’No I don’t either [laughs]’ was a way to establish empathy and encourage the interviewee to engage with the questions about the ‘impact of gifts’. Immediately after saying this, the interviewer included herself as one of the research team (‘We are trying to find out…’) and gave the desired explanation of what the questions meant, which then elicited a codable response.

**Fig. 1:**
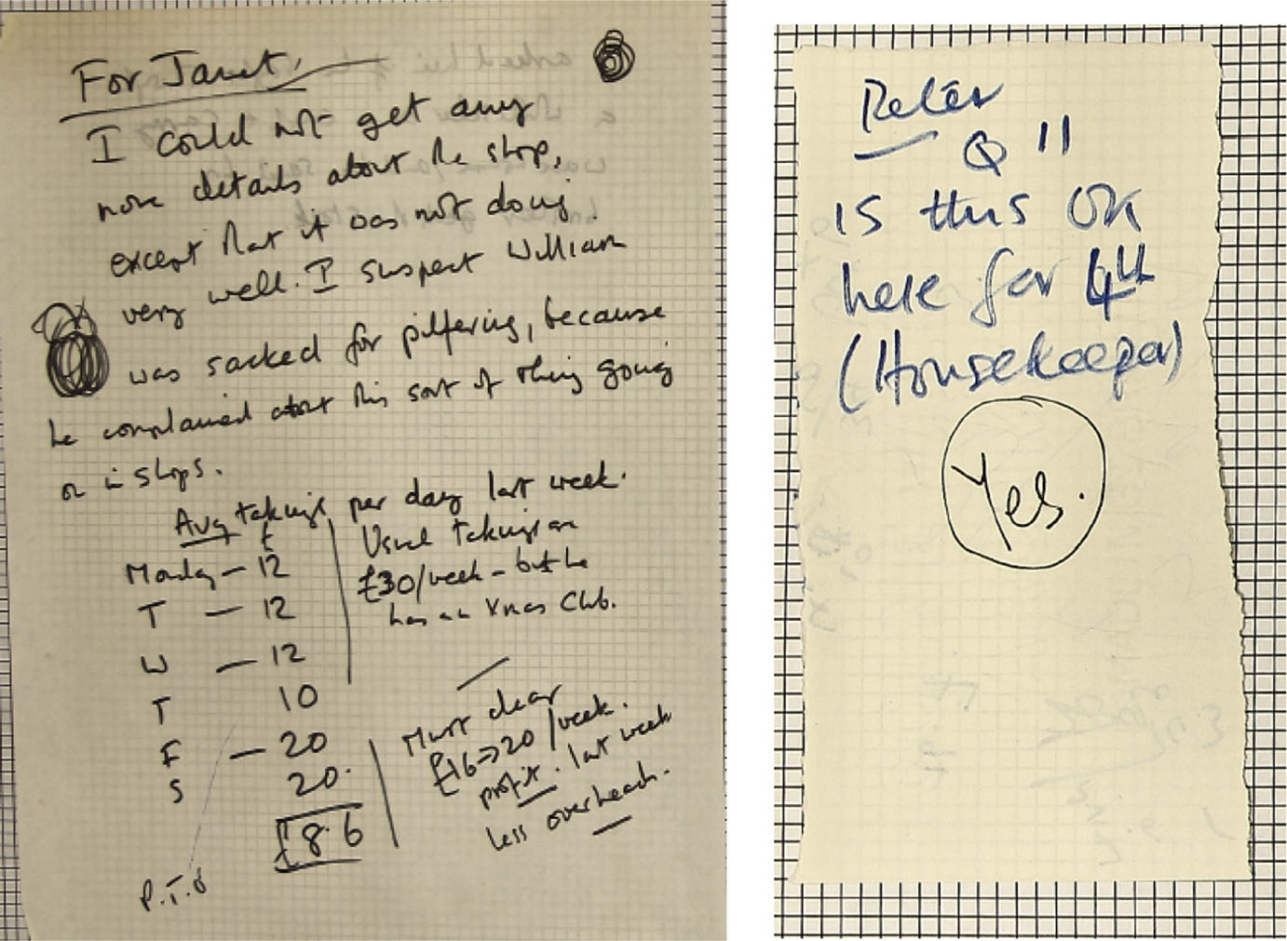
Notes inserted into survey booklets by field interviewers. On the *left*, a note from a field interviewer to a supervisor. On the *right*, a query about coding for question 11 of the survey for Peter Townsend and his reply

One original feature of the study reported here is that it provides a rare example of transcribed survey interviews. The PSE transcripts showed how the interviewers experienced asking questions and made coding choices, regardless of whether they wanted to convey anything to the survey managers. In contrast, in the PinUK survey, field interviewers took the booklets home and augmented or completed any notes they wished the research team to see. Figure 1, on the left, provides a typical example of these (see Phoenix et al. 2017, forthcoming, for in-depth analyses of other booklet marginalia examples). If they chose not to do this, the research team would never know how the interviewers found the interview. Indeed, these micro-paradata were important for Townsend’s later analyses published in *Poverty in the United Kingdom* (1979), for example forming the case studies on the impact of poverty that are the basis for Chapter 8 and those on the rich that inform Chapter 9. In the later PSE survey the more limited possibilities for deliberate communications with the central research team and freedom to be creative in pursuing questions arguably removes some possibilities for social interaction between field interviewer and interviewee and for independent employment of skills. This does not mean, however, that contemporary field interviewers do not have to work hard to find a way to produce the required survey data, an issue we will discuss later.

A further important change wrought by technological development is that there was one material copy of a PinUK survey booklet, which the field interviewer gave to the central team of researchers (or to a supervisor who then passed it back). The university-based team sometimes also inserted their own comments on these booklets in different colours of ink, or on inserted pieces of paper, in dialogue with the field interviewers’ paradata (as in Fig. 1, on the right). In contrast, the fact that research team members all work with a virtual copy of contemporary surveys means that the opportunity is lost to see at a glance which members of the team have worked on the interview and which questions elicited discussion and negotiation amongst the team. The digital files provide less opportunity to see the research as collaborative work and, arguably, to develop understanding of what is difficult for field interviewers.

## Social change and social survey research in iterative relationship

Townsend was a public sociologist committed to campaigning against poverty, inequality and injustice (Bailey 2014; Sinfield et al. 2011). As with all his research, the PinUK survey was intended to fuel social change. The method of measuring poverty that Townsend developed for PinUK was both rigorous and political, in challenging élite and detached ideas about what indicated and constituted poverty (Mullen 1987)—part of the shift away from the differentiation and classification of gentlemanly social science noted earlier. Between the PinUK and PSE surveys, another marked social change is that researchers are now expected to pose the aims of their research in ‘objective’, depoliticised terms, while methods are treated more as technical skills to be honed, abstracted from topic and social context (Brannen and Edwards 2007). Thus, despite increasing epistemological emphasis on co-construction and reflexivity in qualitative research, contemporary survey researchers do not foreground their views on poverty when they seek funding and conduct research (though they may indeed be politically committed). So while research increasingly is intended to produce social impact, researchers are less likely to be explicit about intended political social change outcomes as a rationale for research.

Since the 1960s there have been shifts in the kinds of survey questions that are deemed acceptable. In the 1960s, privacy boundaries were more strictly drawn around asking about financial matters. The PinUK survey instructed field interviewers to estimate the value of owner-occupied property rather than ask directly, and some of the micro paradata on the PinUK booklets tries to collate the evidence the field interviewer has amassed on household income or expenditure. This is sometimes a difficult task. For example, one survey booklet is characterised by scribbled, crossed-out and recalculated figures along with comments such as ‘*Tax position very confused*’ and ‘*Almost impossible to obtain simple answer to anything!*’ Against a question about housekeeping and board the field interviewer writes to the research team: ‘*I hope you can make more of this than I can* –’ (Booklet 2/10 2353). Several of the field interviewers we video interviewed recalled how common it was for wives in the 1960s to be unaware of how much their husband earned. Even simple questions about financial affairs, such as the amount of pension received each week, sometimes provoked angry responses, as noted in this marginal micro paradata:

Showed great anger at being asked these personal questions and said that if I wanted such information I could get it from the authorities. I tried everything I knew to try to calm him down but had to leave this section … sorry could not get more detail but it was tricky. (Booklet 9/48 2101)

In recent surveys it appears that income is easier to ask about; none of the PSE interview questions about income provoked anger even where respondents refused to answer them. Sex now seems to be harder to ask about than in the late 1960s, however. One PinUK field interviewer (AC) recollected that some women offered details of their sex lives even though she was not asking about them: *“*[*The women*] *would tell you every dot and comma about their sex lives that you didn*’*t really want to know, but they would not tell you anything about their finances.”* (also cf. Martin 1990: 2). The PSE team decided a validated question module about sexual violence was ‘too graphic’ for a survey of this nature and asked less detailed questions instead.

Race shows another shift in social sensitivities. As was the convention at the time, the PinUK survey booklet instructions to field interviewers assumed that direct questioning was likely to cause offence and relied on the interviewer’s observation of ‘colour’.^6^ The later PSE survey follows other current surveys in using the Census ethnic group classification and asking for self-identification is seen as unproblematic. The Census did not include any questions on race until the 1980s. By contrast, issues about responsibility for doing housework have become more sensitive. In the PinUK survey, such questions were not considered to be problematic and it was taken for granted that there was a clear gendered division of household labour. For example only ‘housewives’ were asked if they felt tired. However, in the PSE survey, questions about how many hours household members spent each week doing ‘cooking and housework’ were moved to the self-completion section after the survey pilot as field interviewers feared that these questions might provoke arguments and possibly domestic violence.

Mores about what can and cannot be asked directly may reflect social norms around public and private information. They may also contain traces of past understandings, prior to mass democratisation and assumptions of rational citizenry, that the general population is not able to speak for themselves on some matters or to be trusted to do so and, as a result, relied on the field researchers and professional practitioners as observers (as with Booth’s poverty survey of London).

This disparity between the surveys in what can be asked, and how, provides a reminder that analyses of social surveys have to situate findings in the context of normative expectations about what is askable and what generally is silenced as private in particular contexts. In relation to the history of poverty research in particular, the questions currently asked in surveys reflect reformulations of the criteria for measuring poverty that are largely due to the methodology developed for the PinUK survey; away from abject poverty through relative definitions to broader understandings of the causes and consequences of poverty as well as to recognition that household poverty has differential impacts on its members by age, generation and gender (e.g. Brannen and Wilson 1987). Laying out these differences between the surveys side by side helps to illuminate reasons why social surveys, cohort studies and censuses cannot ask exactly the same questions over time, but must partly be forged anew.

Differences in what can be asked and how are compounded by shifts in power relations and ethical expectations. The PinUK survey, for example, required a depth of detail that required large amounts of time to be invested by both field interviewer and informant. An average interview could take 2.5 h, depending on the number of people in the household (the Booklet 2/10 2353 interview mentioned above amounted to 7 h over three visits). Today, for reasons both of economisation and expectations about interviewee tolerance, survey interviews often view an hour as an upper limit. This shift in the time spent signals a shift in power relations between interviewee and interviewer, (where the former now is unlikely to feel deference towards the latter), as well as the foregrounding of ethics through ethical codes and institutional clearance. For example, it is common practice for experienced field interviewers to be employed to convert refusals to interviews (Sturgis and Campanelli 1998). The marginal paradata in some of the PinUK survey booklets, however, indicate that ‘converters’ might go to greater lengths to get interviews than would be considered acceptable today. For example, one wrote on the first page inside the survey booklet:

This woman was disabled, and very nervous. I caught her coming to the door to a neighbour, and interviewed in hall. She got very tired of the questions and one/two were genuinely beyond her. I think like the other interviewer, I would have had great diff. [sic] in getting her to answer the door if I had not been lucky. (Booklet 6/35 1309)

The above extract of micro paradata raises issues of ‘informed consent’ and the quality of data produced from people who are unwilling to take part in the study, that ethics committees would be reluctant to approve.

The PinUK survey booklets included a space for field interviewers to make comments and reflections, headed: ‘*Please write in any additional notes*’. This produced many instances of marginal paradata that make clear that the field interviewers felt empowered to treat their ‘informants’ as social human beings and so sometimes spent time with them beyond the needs of the survey, or wrote micro paradata that showed their empathy with those living in impoverished conditions. This sometimes entailed field interviewers acting in ways that would now be considered unethical, even if caring and friendly. For example, one field interviewer wrote that he had left after tucking up in bed his ‘old lady’ interviewee who was tired out by the survey question marathon, while another bought some bottles of beer and returned to share these over an evening meal with a lonely interviewee.

The central team led by Townsend also occasionally inserted notes or letters into survey booklets that revealed that they had intervened to ameliorate interviewees’ circumstances in liberal humanist ways. Examples include approaching a race relations organisation on an informant’s behalf, or sending a ‘grant’ to a mother recorded by the field interviewer as not having eaten for several days so that she could feed her child. Situations such as parents skipping meals in favour of feeding their children are still evident for those living in poverty today (Gordon et al. 2013; Purdam et al. 2014), though the response of survey research teams is unlikely to be (overtly) the same. In contemporary surveys, in what is sometimes seen as a ‘posthumanist’ period (e.g. Raffensøe 2013), empathy is now more hidden. Currently, field interviewers are aware that the time they spend on particular questions is scrutinised, which has implications for how much time they spend on areas that preoccupy interviewees. Our analysis of the transcripts of the PSE audio-recorded interviews shows similar resourcefulness on the part of the field interviewers^7^ as shown in the paradata written by the PinUK interviewers. One key difference here is that, while the PinUK field interviewers could, and often did, make apparent in their micro paradata notations, those of the PSE interviewers would remain largely invisible to the research team were it not for the audio-recordings for this project. Further, the PSE transcripts were better able to show the process and strategies interviewers employed to keep participants engaged in the interview while also following the protocol (Bell et al. 2016). In the course of gathering the required data for the survey, the field interviewers variously provided interviewees with emotional support, advice or information, complimented them, made jokes, and/or revealed limited personal information.

Moving from one question to another in the formal and standardised manner encouraged in survey organisations’ training could prove difficult or, arguably, unethical. PSE interviewers often demonstrated skills in balancing the pace and requirements of the survey questions with the research relationship; skills that for the most part are invisible (other than through audio-recordings). In the following extract, the field interviewer did not become emotionally engaged when painful information was revealed at various points in the interview, but acknowledged and engaged with the issues, dwelling on neutral or positive aspects, rather than moving straight on to the next question:

Interviewer: Okay. Now are you able to see that? This is self-completion. I can do it.

Interviewee: No.

Interviewer: Okay, I’ll read it out.

Interviewee: My left eye’s very poor so I have to rely on just this eye.

Interviewer: Is that as a result of your injuries?

Interviewee: Yeah.

Interviewer: What happened then?

Interviewee: I got assaulted, walking past a pub… It was attempted murder apparently.

Interviewer: Really?

Interviewee: Yeah. I had to have all me face reconstructed and have to have–

Interviewer: They’ve done a good job cos you can hardly tell.

Interviewee: …They broke all my eye sockets, I had to have bars screwed into me head here, with a frame on, to hold me face together… It’s horrific.

/…/

I Interviewer: God, they’ve done an amazing job I have to say.

While expressing sympathy, the interviewer does manage to curtail the discussion: **‘** *Right, I*’*m sorry to… Right, let me do, I*’*ll do this self*-*completion for you…*’ This skilful handling forms a contrast to another interview, where a different field interviewer, informed by the interviewee that her niece had been murdered responded with ‘*Right*’ and moved on, maintaining the standardised, consistent survey interaction, but without apparent sympathy or note. Indeed, Fowler and Mangione (1990) have suggested that interview standardisation could constrain the development of rapport between interviewer and respondent (see also Bell et al. 2016 for discussion of this issue).

It was certainly not the case that all of the PinUK booklets we analysed for paradata showed evidence of interviewer sympathy. The point here, however, is that, even if they are not told to do so, the different conditions of knowledge production made it more likely that interviewers would suppress sympathetic reactions in the PSE than in PinUK in attempting to treat each interview situation in a standardised way and so as interchangeable. While it is difficult to gain comprehensive evidence on this. This was agreed to be the case by the project Community of Interest group, composed of senior researchers and managers of major contemporary surveys (discussion 1.8.14).

## The intersection of technological, social and professional role change

The technical and social shifts discussed above have produced changes in the nature of the professional role available to social survey field interviewers. At first sight little appears to have changed between the poverty surveys of 1967/1968 and 2012. In both instances the field interviewers are not foregrounded in the research teams and in publications from the projects. Most are employed on contracts, only to do the interviews, and are at the bottom of the research hierarchy. However, in PinUK, the field interviewers are visible in the paper survey booklets, embodied through their names written on the top inside page of the booklet and their handwritten notes on codes and especially if they wrote marginalia. On PSE, the field interviewers are disembodied and absent from the digitally recorded data (except in rare studies such as ours).

The meaning of ‘professionalism’ for a field interviewer has transformed, from the exercise of individually tailored interpersonal skills to the deployment of standardisation. Historically, ‘professional’ interviewers have been valued for the conduct of survey research. Rowntree recorded that he appreciated their persistence in paying ‘*many thousands of visits*’ and for their exercise of ‘*no small amount of discernment and tact*’ (Rowntree 1902: 14, quoted in Platt 2014: 35). The PinUK field interviewers were also encouraged to deploy discernment and tact. For example, an instruction in the survey booklet for a question about daily activities states:

It would be insensitive and unnecessary to ask questions about the daily activities of the bedfast … The same is true of any situations in which the questions are likely to cause great distress.

The field interviewers were also required to exercise their own observational judgements, as noted above in relation to ‘colour’. In other ways, however, survey field interviewers of that period have been judged a non-professional group. The majority were married women seeking part-time employment (Rossi et al. 1983), and Morton-Williams has described them as ‘*the Cinderella of the survey industry … undertrained, undersupervised, underpaid and undervalued*’ (1993: 3). Since then, there has been a decline in the role of survey interviewer as observer (Converse 2009), and the position has become professionalised. The main thrust of this professionalisation has been rigorous training with an emphasis on improving consistency between interviewers’ approaches. Further, field interviewers are now paid more than ‘pin money’ and many undertake the role as a full time career. Indeed, a diminishing pool of people willing to take on the low-paid work of field interviewing combined with the increasing professionalisation of the role through training led to a rise in payment—and fed into the push towards economisation in the face of soaring survey costs (Community of Interest discussion 1.8.14).

There is also greater segregation between the core research team and the field interviewers now (Morton-Williams 1993). Most of the PinUK field interviewers were recruited by, and worked directly to, the core research team, linked through supervisors rather than through a social survey agency (apart from two ‘special’ areas where an agency was employed). This situation was unusual, even for the 1960s. Direct recruitment specifically to work on PinUK may have contributed to some of the field interviewers’ confidence that they could write micro paradata of various sorts to the central research team, and meant that some recorded their own standpoints on poverty and family life and/or evaluations of informants’ characters or their claims (Phoenix et al. 2017). It is important to note that field interviewers from the market research company were less likely than the directly appointed field interviewers to write marginalia; dissimilar conditions of employment and expectations may in themselves produce different commitment to the research. Indeed, in a summary progress report for the Rowntree Trust dated September 1968, Townsend records that he aimed to recruit field interviewers who were ‘*genuinely interested in participating in the research and willing to face the challenge of a long questionnaire often involving several interviews with different household members.*’ (p. 3), although he notes that the drop-out rate for his field interviewers was higher than he expected.

The field interviewers’ marginal comments helped to deepen the core team’s understanding of their informants and of poverty in everyday life for a wide range of families. As noted earlier, Townsend drew on these micro paradata to enliven the largely statistical text in his influential book, *Poverty in the United Kingdom* (1979), with narrative case studies. Such evaluations and standpoints are not available to survey research teams currently, particularly since surveys are now almost exclusively conducted by survey agencies, so that field interviewers are not employed by the survey research team.

There are two important ways in which the practice of survey interviewing is now easier and safer than it was half a century or so ago. First, in poverty surveys at least, field interviewers no longer need to spend the long periods of time the PinUK interviewers devoted to calculating household income and outgoings (made even more difficult in cases where information was contradictory and confusing). The instruction to field interviewers about a question addressing employee’s pension contributions, for example, reads:

When given a percentage note that it may be calculated on basic wages rather than earnings and you should note this so that we can adjust the figure in the office. Estimate the proportion of normal earning the previous contribution amounts to—correct to nearest percentage point.

The tedium of entering figures, calculating totals, doing percentages, and checking them in the office has been replaced by automatic computer calculation. Additionally, Computer Aided Interviewing allows sensitive questions, which could provoke an embarrassed or angry response, to be asked in a self-completion section to which the respondents know the field interviewer has no access. This is potentially both less embarrassing for the interviewees and easier for the field interviewer who does not have to make great efforts to achieve a response.

Secondly, along with a lack of standardised training went a lack of protection for field interviewers on the PinUK survey. They travelled long distances to unfamiliar neighbourhoods to interview people in households and could be away from the office for days without anyone keeping close track of where they were and when they returned. Today, as part of the rigorous training they receive, issues of safeguarding for field interviewers are on the agenda. This aspect of the ‘electronic leash’ and professionalisation is certainly preferable to the laissez-faire approach to interviewers’ safety that pertained half a century ago.

## Conclusion

This article has used examples from two different kinds of qualitative paradata to indicate how technical, social and professional role changes in the process of conducting research into poverty over the 45 years between the innovative conception and measurement of poverty marked by Townsend’s 1967/1968 Poverty in the UK survey, and the 2012 Poverty and Social Exclusion in the UK survey, have affected the relations of production of data, especially for the field interviewers. We have highlighted and reflected upon the intrinsic, particularistic and relational nature of data gathering through face-to-face interviews, that we have shown pertains as much for current survey researchers as for earlier ones (despite contemporary attempts to override this and standardise the process). We have shown how wider technical, social and professional shifts also play a part in shaping these relations and interviewing practices. Drawing on micro paradata from the two surveys, respectively as written and audio-recorded ‘by-products’, we have explored how these changes, and some continuities, are shot through with the three main characterising trends in post-war social survey research: economisation, institutionalisation and professionalisation.

Economisation has been pursued in an attempt to deal with the burgeoning costs of conducting social surveys, aided by technological changes and driven in part by the increasing remuneration of survey field interview work as it has become professionalised. The length of time that a survey interview lasts has reduced, not just because of economisation but also linked to social changes that have shifted power relations between field interviewers and survey respondents and the decreasing tolerance of the latter. Indeed survey response rates have fallen since the 1960s (Dunn and Moore 2014). Along with a shift away from willingness to participate in survey research, the introduction of institutional ethical governance has also meant that contemporary field interviewers on the PSE survey may find it harder to ‘convert’ refusals to participate in a survey into assent than did the PinUK field interviewers, who sometimes used tactics that would not now be condoned (as discussed above).

Greater institutionalisation of survey research, in the form of organisational consolidation, has produced a push for standardisation and consistency in the data collection process. Along with moves towards explicit, rigorous training, methods of field interviewing have become less interpersonal and more ‘scientised’ as skills to be learned.

Professionally, the ‘electronic leash’ enabled through technological change is a major change from field interviewers being out on their own with paper and pen. CAPI means that codes and pathways through questions are predetermined. Unlike the PinUK survey booklet, there is no dedicated, open space for field interviewers to ‘talk to’ the central research team about anything they wish (and not just codes or answers to particular questions). There has been a shift away from valuing the field interviewers’ observations as data to a stress on standardisation in eliciting data from the respondent responses. Thus the confluence of economisation, institutionalisation and professionalisation has meant that the labour involved in the production of poverty survey data becomes less visible. At the same time, political commitment on the part of researchers has also became invisibilised, with impact now being an ostensible contemporary goal, but of a different order to the overt commitment to social change that underpinned Townsend’s PinUK survey.

Across the 45 year period between PinUK and PSE, field interviewers have remained at the bottom of research hierarchies, but there was more ‘space’ for in the 1967/1968 survey for them to make their labour and efforts visible to the central research team. In general, the history of survey research illuminated through our reflection on micro paradata from the PinUK and PSE surveys is one of decreased independence and increasing invisibilisation: of political commitment, of field interviewer labour, and of the social and humanistic relations of survey data production. There have, however, certainly have been gains for poverty survey field interviewers over the years. In particular, they are paid more as a profession, are spared the tedium of calculations, and are more safeguarded.

We note that there has been a shift from an élite to a mass democratised society, with a decrease in deference and willingness to participate in surveys. In these circumstances contemporary field interviewers may have to work hard to establish rapport and commitment through focused relational interview work, because they cannot rely on a sense of deference to secure information in the way that the PinUK interviewers frequently could. Ironically, therefore, current societal norms and relations mean that contemporary survey interviewers may well need to practice targeting particular interactions to particular interviewees in order to secure and maintain a survey interview more than was necessary in the past. It seems, however, that this need is now regarded as undesirable in survey interview practice and is more obscured than it was in the past.

## Footnotes

1 Platt (2014) asserts that the researchers who worked with Charles Booth in his late nineteenth/early twenteenth century survey into life and labour in London (widely considered to be ‘the start’ of the social survey and laying the foundation for poverty studies) were one of the greatest legacies of his study (e.g. Beatrice Potter/Webb, Octavia Hill and Hubert Llewellyn Smith). The same may be said of the researchers who conducted Townsend’s pilot studies: Hilary Land, now Emeritus Professor of Family Policy and Child Welfare, University of Bristol; Dennis Marsden became Professor of Sociology, University of Essex; Adrian Sinfield is Emeritus Professor of Social Policy, University of Edinburgh; and John Veit Wilson, Visiting Professor of Sociology, Newcastle University and Emeritus Professor of Social Policy, Northumbria University.

2 Other research on which PSE builds includes the 1983 and 1990 Breadline Britain surveys (Mack and Lansley 1985; Gordon and Pantazis 1997), the 1999 Poverty and Social Exclusion Survey (Gordon et al. 2000; Pantazis et al. 2006) and the 2002/03 Northern Ireland Poverty and Social Exclusion Survey (Hillyard et al. 2003).

3 Ethical approval for the project was received from the University of Southampton (ERGO ID 6043).

4 The PSE survey also included 3551 interviews where data on one or more household members was partial or incomplete.

5 The PinUK main survey interviewed 2052 households (6045 people) and the follow-up survey of ‘special’ areas interviewed another 1514 households (3539 people). Unfortunately 961 of survey booklets from the ‘special’ areas were not archived in the UK Data Service, presumably having been lost or destroyed at some stage, so we have been unable to digitise them.

6 In the 1970 s and 1980 s the General Household Survey field interviewers assessed from observation only, if each member of the household was ‘Coloured’ or ‘White’ (variable name COLOUR). The instructions were to ‘*Code according to your own observation; but you include as coloured all persons who would not be described as white—e.g. Negroes, Indians and Pakistanis, Chinese and Japanese.*’

7 The sample of 23 survey interviews was selected by the administering organisation, NatCen. All the field interviewers were experienced and agreed to participate.
